# Tacrolimus–Sirolimus Combined Exposure and Acute Rejection in Kidney Transplant Recipients Undergoing Early Conversion to Sirolimus: A Multicenter Retrospective Cohort Threshold Analysis

**DOI:** 10.3390/jcm14217808

**Published:** 2025-11-03

**Authors:** Byunghyun Choi, Youngmin Ko, Jin-Myung Kim, Hye Eun Kwon, Young Hoon Kim, Sung Shin, Joo Hee Jung, Hyunwook Kwon

**Affiliations:** 1 Division of Hepato-Biliary-Pancreatic Surgery and Transplantation, Department of Surgery, Pusan National University Yangsan Hospital, Pusan National University School of Medicine, Busan 43241, Republic of Korea; gmoolpop@gmail.com; 2 Division of Kidney and Pancreas Transplantation, Department of Surgery, Asan Medical Center, University of Ulsan College of Medicine, Seoul 05505, Republic of Korea; ymko@amc.seoul.kr (Y.K.);

**Keywords:** kidney transplantation, immunosuppressive agents, calcineurin inhibitors, sirolimus, graft rejection

## Abstract

**Background/Objectives**: Combining calcineurin inhibitors (CNIs) with mTOR inhibitors has been explored to reduce CNI exposure. However, the safety of this early conversion approach remains uncertain, and the optimal therapeutic targets for tacrolimus and sirolimus trough concentrations in patients have not been clearly established. **Method**: In this retrospective multicenter cohort, we analyzed 8027 kidney transplant recipients and compared a standard group (tacrolimus + MMF) with an early conversion group (MMF to sirolimus within 3 months post-transplant). To address group-size and baseline imbalances—including differences in age, induction therapy, and diabetes—we performed 4:1 propensity score matching, yielding a cohort of 1180 patients. The primary endpoint was biopsy-proven acute rejection between 3 and 12 months post-transplant. **Results**: The early conversion group had a higher acute rejection rate (7.6%) than the standard group (2.9%; *p* = 0.001). Stepwise threshold analysis suggested a combined tacrolimus–sirolimus exposure (Tacro–Siro Csum) of 11.6 ng/mL as the level associated with the lowest rejection risk, whereas levels < 8.5 ng/mL were substantially higher risk. Patients with Tacro–Siro Csum < 8.5 ng/mL showed a higher rejection rate even when CNI trough levels were adequate (*p* = 0.031). Tacro–Siro Csum showed the strongest inverse correlation with rejection (r = −0.33), underscoring its utility as a composite indicator. **Conclusions**: In early sirolimus conversion, the combined trough level of tacrolimus and sirolimus is more important than either drug alone. To reduce the risk of acute rejection, it is crucial to maintain this combined concentration at a therapeutic level.

## 1. Introduction

The standard immunosuppressive regimen most widely used in kidney transplantation is triple therapy—tacrolimus, mycophenolate mofetil (MMF), and corticosteroids [1]. Calcineurin inhibitors (CNIs) are central to preventing acute rejection, but their use is associated with hypertension, nephrotoxicity, and graft fibrosis [2]. Multiple studies have evaluated CNI replacement with alternative agents, but these strategies have not achieved comparable acute rejection rates [3,4]. Consequently, mTOR inhibitors have not proven suitable as complete CNI substitutes. Trials have therefore focused on reducing CNI exposure by combining CNIs with mTOR inhibitors [4,5]. Concomitant use has shown potential benefits, including preservation of graft function, a lower infection incidence, and possible antineoplastic effects [6,7,8]. Because mTOR inhibitors impair wound healing, initiation after wound healing is considered safer [9,10].

In recipients undergoing early conversion from MMF to sirolimus, optimal tacrolimus and sirolimus trough targets remain uncertain. No prior study has established how to adjust and maintain these levels—either individually or in combination—or identified a consensus combined trough exposure (Csum) that balances efficacy and safety during the first post-transplant year, when rejection risk is highest.

This study aimed to define the individual therapeutic ranges of tacrolimus and sirolimus and to determine whether their combined trough level can achieve an acute rejection rate comparable to the standard triple regimen using data from a large Korean kidney transplant cohort.

## 2. Materials and Methods

### 2.1. Patients

This retrospective cohort study analyzed data collected between January 2005 and December 2020 from five kidney transplant centers in Republic of Korea that participated in a prior multicenter study [11,12]. Four centers with complete sirolimus trough concentration data were included in the analysis. Adult kidney transplant recipients (≥18 years) with at least 1 year of post-transplant follow-up were eligible. Patients who received multiple kidney transplants or concomitant non-renal solid organ transplants were excluded. Based on these criteria, 9992 recipients were initially identified. Of these, 896 patients who discontinued CNIs within the first post-transplant year and 1069 patients who received cyclosporine-based regimens were excluded, resulting in a final cohort of 8027 patients. These exclusions were made to maintain pharmacologic consistency for exposure–response analysis, as cyclosporine has different immunosuppressive potency and pharmacokinetic characteristics compared with tacrolimus. The study cohort was categorized into two groups: (1) the standard group, comprising recipients who maintained tacrolimus plus MMF throughout the first post-transplant year; and (2) the early conversion group, comprising recipients converted from MMF to sirolimus within the first 3 months after transplantation who then maintained tacrolimus–sirolimus therapy for at least 1 subsequent year. To address the substantial difference in group sizes, 4:1 propensity score matching (PSM) was performed.

### 2.2. Definitions

Complete trough-level data were defined as at least three separate trough concentration measurements within the first post-transplant year. The combined trough concentration of tacrolimus and sirolimus (Tac–Siro Csum) was calculated by summing both drugs’ concentrations at each time point and averaging these values over the first post-transplant year, as follows:

$$Csum_{i}=\;\left(\frac{1}{N}\right)\Sigma_{t=1}^{N}\left(Tac_{\left\{i,t\right\}}+\;Siro_{\left\{i,t\right\}}\right)$$

where *N* is the number of paired trough concentration measurements available for patient *i* between 3 and 12 months post-transplant.

This metric was used as a surrogate of overall immunosuppressive exposure in the Tacro + Siro group. The primary endpoint was biopsy-proven acute rejection between 3 and 12 months post-transplant, i.e., after treatment-group divergence. Most centers used Chemiluminescent Microparticle Immunoassay (CMIA) for routine trough monitoring, while Liquid Chromatography–Tandem Mass Spectrometry (LC–MS/MS) was selectively performed for specific clinical or confirmatory purposes. Desensitization was defined as the administration of rituximab, plasmapheresis, or intravenous immunoglobulin before transplantation due to ABO or HLA incompatibility. BK virus positivity (BKV-positive) was defined as a quantitative plasma PCR result ≥ 3 log copies/mL.

### 2.3. Statistical Analysis

Categorical variables were compared using chi-square or Fisher’s exact tests, as appropriate. Continuous variables were analyzed using Student’s *t*-test. PSM used a logistic regression model including baseline covariates: age, sex, body mass index (BMI), diabetes mellitus, pre-transplant dialysis status, induction therapy, panel-reactive antibody (PRA) class I and II levels, desensitization status, donor age, ABO incompatibility, pre-transplant flow cytometric crossmatch results (B and T cells), and BK virus infection status at 3 months post-transplant. Nearest-neighbor matching without replacement was applied with a caliper of 0.2 standard deviations on the logit of the propensity score. After matching, baseline characteristics were well balanced; recipient age remained imbalanced (standardized mean difference [SMD] = 0.39). To determine the optimal Tac–Siro Csum cutoff, we compared rejection rates across exposure thresholds (7–14 ng/mL) and plotted the corresponding *p*-values on a log scale to visualize trends. Additionally, Pearson correlation was used to assess linear relationships between exposure metrics and rejection risk, with results displayed as a correlation heatmap.

All analyses were conducted in Python (v3.11.8). The pandas (v1.5.3) and numpy (v1.24.0) packages supported data preprocessing and transformation. PSM was implemented with statsmodels (v0.13.5) using a logistic regression model with nearest-neighbor matching without replacement and a 0.2-SD caliper on the logit of the propensity score. Key visualizations, including boxplots and *p*-value trend plots across tacrolimus–sirolimus exposure thresholds, were generated with matplotlib (v3.6.3). Pearson correlation heatmaps depicting relationships between immunosuppressant levels and rejection were produced with seaborn (v0.11.2).

## 3. Results

### 3.1. Patient Demographics and Clinical Characteristics

We analyzed 8027 kidney transplantation recipients, including 7791 (97.1%) in the standard group and 236 (2.9%) in the early-conversion group. Relative to the standard group, the early-conversion group was older (49.2 ± 12.2 vs. 45.4 ± 13.9 years; *p* < 0.001) and had a higher prevalence of diabetes (27.5% vs. 20.4%; *p* = 0.009). Induction therapy also differed (*p* < 0.001), with less frequent basiliximab use (63.6% vs. 77.0%) and more frequent anti-thymocyte globulin use (30.1% vs. 19.7%) in the early-conversion group. BK virus infection at 3 months was higher in the early-conversion group (16.9% vs. 8.9%; *p* < 0.001) (Table 1).

After 1:4 PSM, 1180 kidney transplant recipients were included: 944 (80.0%) in the standard group and 236 (20.0%) in the early-conversion group. All baseline variables—including sex, BMI, diabetes, desensitization, PRA levels, HLA mismatch, donor age, preemptive transplantation, ABO incompatibility, pre-transplant DSA, and 3-month BK virus infection—had SMDs < 0.1, indicating good balance, except recipient age (SMD = 0.39). Rejection between 3 and 12 months post-transplant occurred more often in the early-conversion group (7.6%) than in the standard group (2.9%), with SMD = 0.21 and *p* = 0.001 (Table 2).

### 3.2. Risk of Acute Rejection by Combined Drug Exposure

**Figure 1:**
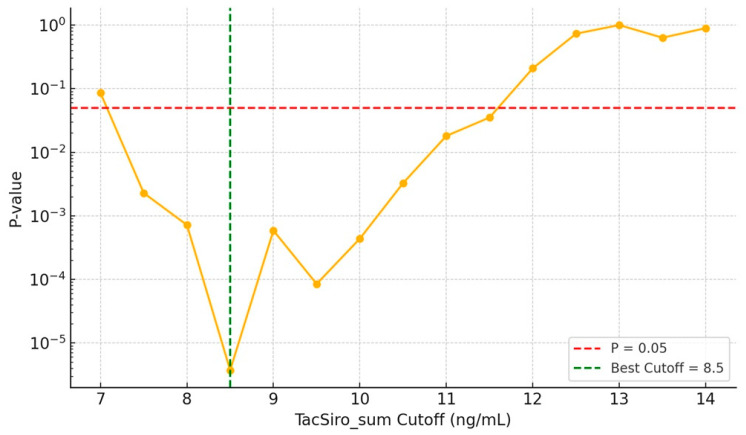
Acute rejection rate by combined tacrolimus–sirolimus trough levels. Bar graph of acute rejection rates stratified by the cumulative sum of tacrolimus and sirolimus. Yellow dots indicate the *p*-values computed at each incremental cutoff. The yellow line visualizes the trend. The red dashed line denotes the *p* = 0.05 reference. The green dashed line marks the cutoff (8.5 ng/mL) where the separation between the two groups was most pronounced. Rejection risk remained significantly elevated below 11.6 ng/mL, with the strongest risk separation at 8.5 ng/mL. Concentrations ≥ 11.6 ng/mL appeared safest, whereas levels < 8.5 ng/mL were associated with substantially increased rejection risk.

Figure 1 shows the stepwise threshold analysis of Tac–Siro Csum in relation to acute rejection. Rejection risk remained significantly elevated below approximately 11.6 ng/mL, with the strongest risk separation at 8.5 ng/mL. Concentrations ≥ 11.6 ng/mL appeared safest, whereas levels < 8.5 ng/mL were associated with markedly increased rejection risk.

**Figure 2:**
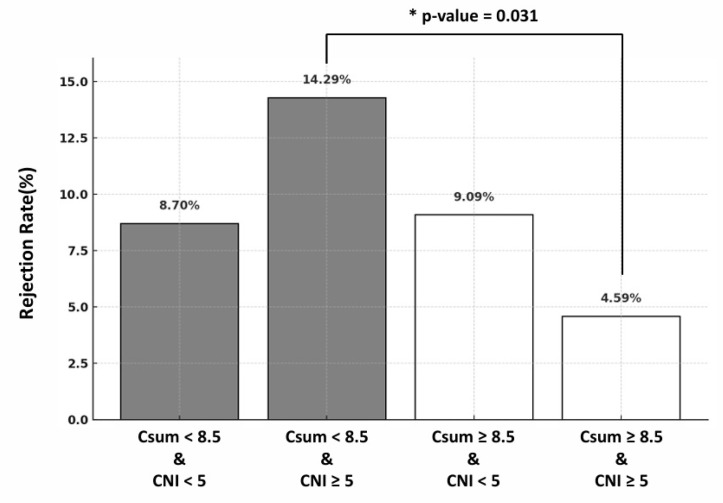
Subgroup comparison of acute rejection according to 1-year mean tacrolimus trough and Csum levels. Recipients undergoing early MMF-to-sirolimus conversion were classified into four groups by mean 1-year tacrolimus trough (≥5 vs. <5 ng/mL) and Csum (≥8.5 vs. <8.5 ng/mL). Grey bars indicate Tac–Siro Csum < 8.5 ng/mL, and white bars indicate Tac–Siro Csum ≥ 8.5 ng/mL. The Csum < 8.5 with tacrolimus ≥ 5 group had the highest rejection rate; the group with both higher Csum and tacrolimus levels had the lowest; * indicates *p* = 0.031.

We then assessed rejection rates by Tac–Siro Csum and CNI trough levels (Figure 2). The highest rejection rate (14.3%) occurred with Tac–Siro Csum < 8.5 ng/mL and CNI < 5 ng/mL, whereas the lowest rate (4.6%) occurred when both Tac–Siro Csum ≥ 8.5 ng/mL and CNI ≥ 5 ng/mL. Notably, even with adequate CNI exposure (≥5 ng/mL), low Tac–Siro Csum (<8.5 ng/mL) was associated with a higher rejection rate than sufficient cumulative exposure (*p* = 0.031). These findings suggest that adequate Tac–Siro exposure is critical for preventing rejection, independent of CNI levels.

**Figure 3:**
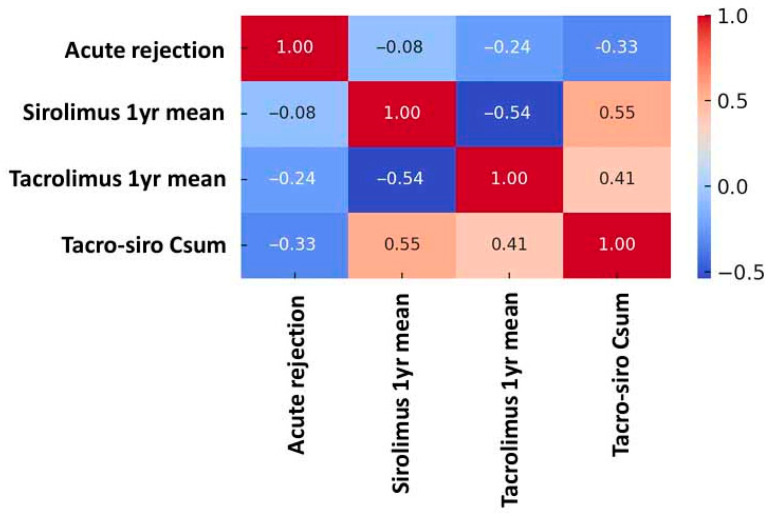
Correlation heatmap of drug exposure and acute rejection. Spearman correlation analysis demonstrated that acute rejection correlated most strongly with the combined tacrolimus-sirolimus level (r = −0.33), with a weaker correlation to tacrolimus alone (r = −0.24). In contrast, the sirolimus monotherapy level showed no significant correlation with rejection (r = −0.08).

Consistently, the correlation heatmap (Figure 3) showed the strongest inverse correlation with rejection for Tac–Siro Csum (r = −0.33), compared with sirolimus (r = −0.08) and tacrolimus (r = −0.24) considered separately, underscoring the utility of Tac–Siro Csum as a composite indicator of immunosuppressive adequacy.

### 3.3. Renal Function and Infection Outcomes by Treatment Strategy

**Figure 4:**
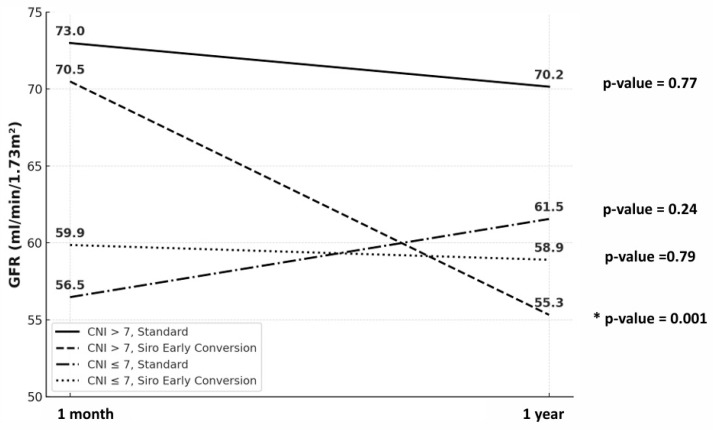
Glomerular filtration rate at 1 month and 1 year post-transplantation by CNI exposure (≤7 vs. >7 ng/mL) and treatment strategy (standard vs. early sirolimus conversion). Within-group comparisons showed a significant eGFR decline only in the CNI > 7 ng/mL with early sirolimus conversion group (* indicates *p* = 0.0014); the other subgroups—CNI > 7 standard, CNI ≤ 7 standard, and CNI ≤ 7 early sirolimus conversion—showed no significant change (*p* = 0.765, *p* = 0.239, and *p* = 0.794, respectively).

Estimated glomerular filtration rate (eGFR) at 1 month and 1 year post-transplantation was evaluated by CNI trough level (≤7 vs. >7 ng/mL) and treatment strategy (standard vs. sirolimus early conversion) (Figure 4). Among the four subgroups, a significant eGFR decline was observed only in the >7 ng/mL CNI with sirolimus early-conversion subgroup (*p* = 0.001). In contrast, the remaining subgroups (CNI > 7 standard, CNI ≤ 7 standard, and CNI ≤ 7 sirolimus early conversion) showed no statistically significant change (*p* = 0.77, *p* = 0.24, and *p* = 0.79, respectively).

We compared infection-related adverse events between the standard regimen and the early sirolimus conversion group (Table 3). Hospitalizations for infection were more frequent with early conversion than with the standard regimen (22.9% vs. 15.5%; *p* = 0.009). The incidence of Pneumocystis jirovecii pneumonia (PCP) did not differ between groups (1.7% vs. 2.3%; *p* = 0.73). BK viremia during 3–12 months post-transplantation was numerically lower with early conversion (4.2% vs. 6.8%) but was not significant (*p* = 0.16).

## 4. Discussion

This study sought to define tacrolimus and sirolimus trough targets that yield an acute rejection rate comparable to standard triple immunosuppression in the first post-transplant year. Neither tacrolimus nor sirolimus trough alone were significantly associated with lower rejection risk versus standard therapy. Instead, cumulative exposure to both agents was the principal determinant, indicating that the combined immunosuppressive effect—rather than either drug alone—drives rejection prevention.

Prior studies have paired mTOR inhibitors with reduced-dose CNIs in kidney transplantation, using different initiation times and trough targets. The TRANSFORM study used a de novo strategy, starting everolimus at transplantation with reduced tacrolimus [13]. Tacrolimus troughs were 4–7 ng/mL for months 0–2 and 2–4 ng/mL for months 7–24, with everolimus maintained at 3–8 ng/mL. In contrast, many early-conversion protocols, including the Korean multicenter RECORD study, initiated sirolimus later and targeted higher sirolimus levels (10–15 ng/mL after postoperative day 14) with correspondingly lower tacrolimus (3–7 ng/mL after 6 months) [14]. A recent Chinese study targeting sirolimus at 5–7 ng/mL and tacrolimus at 3–5 ng/mL also demonstrated noninferiority for efficacy and safety [15]. Despite differences in timing and choice of mTOR agent (everolimus vs. sirolimus), these studies consistently lowered tacrolimus exposure while titrating the mTOR inhibitor to maintain immunologic control. However, guidance on optimal Csum, especially for early-converted high-risk patients, remains lacking. Our study adds to this evidence by examining early MMF-to-sirolimus conversion and providing real-world data on combined trough levels.

Our analyses showed that Tac–Siro Csum below approximately 11.6 ng/mL was associated with a higher risk of acute rejection, with the strongest risk separation at 8.5 ng/mL based on stepwise cutoff and correlation heatmap analyses. Combined exposure, therefore, appears more predictive of acute rejection than CNI trough concentration alone. For immunologically high-risk patients, maintaining Csum ≥ 11.6 ng/mL may be advisable, whereas for lower-risk patients or those requiring overall immunosuppression reduction, maintaining between 8.5 and 11.6 ng/mL could be considered. However, reducing the combined concentration below 8.5 ng/mL within the first post-transplant year may carry a substantially increased risk of acute rejection. However, concurrent sirolimus with high CNI levels (≥7.0 ng/mL), which elevates Csum, was associated with a significant decline in eGFR. This pattern suggests a synergistic nephrotoxicity—plausibly via CNI-related afferent arteriolar vasoconstriction and tubulo-interstitial injury together with mTOR-related podocyte and endothelial effects leading to proteinuria—particularly in vulnerable patients [2,16]. Accordingly, while Csum ≥ 11.6 ng/mL may be necessary for rejection prevention, we advise keeping CNI troughs within a reduced yet effective range (5–7 ng/mL) rather than strictly targeting ≥ 7 ng/mL. In clinical practice, this implies closer surveillance of eGFR, blood pressure, and proteinuria after early sirolimus conversion, with prompt tacrolimus down-titration if kidney function declines. This approach aims to balance the immunologic benefit of higher Csum with protection of graft function, especially in recipients with pre-existing kidney injury [17].

Immunosuppressants are often categorized by their effects on the three signals of T-cell activation. CNIs (e.g., tacrolimus, cyclosporine) target Signal 1, downstream of T-cell receptor engagement and calcium-dependent signaling. Co-stimulation blockers (e.g., belatacept) inhibit Signal 2 by blocking CD28–CD80/86 interactions, preventing full T-cell activation. Agents acting on Signal 3—cytokine-driven T-cell proliferation—include mTOR inhibitors and MMF [18]. Despite both being Signal 3 modulators, these classes act through distinct mechanisms.

MMF inhibits inosine monophosphate dehydrogenase (IMPDH), blocking de novo purine synthesis and limiting proliferation of T and B lymphocytes. mTOR inhibitors, by contrast, block IL-2 receptor downstream signaling, arresting the cell cycle in activated T cells. Although their targets differ, both mTOR and CNIs converge on the IL-2–mediated activation pathway, thereby suppressing T-cell responses [19]. These mechanistic distinctions help explain the superior efficacy of standard triple therapy—CNI, antimetabolite, and corticosteroid—particularly for preventing acute rejection. Replacing the CNI with an mTOR inhibitor has generally not matched rejection outcomes, likely because mTOR inhibition cannot fully replicate the upstream blockade achieved by CNIs [20].

Nevertheless, substituting MMF with an mTOR inhibitor can confer clinical advantages. When paired with reduced-dose CNI and adequate combined exposure (e.g., Tac–Siro Csum ≥ 11.6 ng/mL), mTOR-based regimens may reduce nephrotoxicity relative to conventional CNI-based therapy. Several studies have also reported fewer opportunistic infections, including cytomegalovirus (CMV) and BK virus [16,21,22]. In our cohort, however, infection-related hospitalizations, PCP, and BK viremia did not differ significantly between the standard and early sirolimus conversion groups. This null finding likely reflects the retrospective design rather than randomized allocation. Many patients converted to sirolimus had signs of infection—such as BK virus positivity—at conversion, which likely influenced the decision to switch from MMF. Additionally, both groups contained many recipients at moderate to high immunologic risk, potentially attenuating any antiviral advantage of mTOR-based immunosuppression. Therefore, infection-triggered conversion may have introduced selection bias and confounding in the interpretation of rejection and renal function outcomes. Interestingly, despite higher acute rejection rates, infection-related admissions were also more frequent in the early sirolimus conversion group. This seemingly paradoxical pattern may indicate heterogeneous immune status rather than excessive overall immunosuppression. Because sirolimus conversion was often prompted by infection or intolerance to MMF, patients in this group might have simultaneously faced both impaired immune defense and heightened rejection risk. These findings highlight the challenge of maintaining optimal immunologic balance—preventing rejection while minimizing infection—when modulating combined CNI–mTOR inhibitor exposure.

In addition, the higher prevalence of diabetes among early sirolimus-conversion recipients likely reflects a similar selection bias. Because diabetic patients are at increased risk of infection, CNI-induced nephrotoxicity, and metabolic derangements, clinicians often chose sirolimus conversion to enable CNI minimization. Although propensity score matching was applied to reduce baseline imbalances, this bias cannot be fully excluded, and the two groups should not be regarded as entirely equivalent.

Early postoperative mTOR inhibitor use in kidney transplantation has been linked to higher wound-complication rates [9,10]. Accordingly, initiation is usually deferred until complete wound healing. Although mTOR inhibitors are used to reduce CNI doses, subtherapeutic CNI exposure can increase acute rejection and de novo donor-specific antibodies (dnDSAs) [23]. Our data indicate that combining mycophenolate with an mTOR inhibitor—rather than replacing one with the other—may confer additional immunologic benefit. This putative synergy warrants prospective evaluation [24].

This study had several limitations. First, the retrospective design limited control of confounding variables. PSM reduced baseline imbalances between the standard and early-conversion groups, yet the conversion cohort was small, lowering statistical power and generalizability. Second, the multicenter design prevented uniform data collection across sites; for example, CMV infection and dnDSA data were often missing and were excluded from analysis. Third, sirolimus conversion was frequently triggered by intercurrent complications (e.g., CMV or BK virus infection) rather than pre-planned. Although we adjusted by including BKV positivity at conversion in the PSM model, residual confounding is likely. Thus, baseline risk may have differed between groups. Fourth, trough level assays were performed using both CMIA and LC–MS/MS across participating centers, and subtle inter-laboratory variability may have influenced absolute concentration values. Nevertheless, these differences were likely minimal, because each patient’s Csum was derived from repeated serial measurements, thereby averaging out random analytical and inter-center variations.

## 5. Conclusions

This study suggests that the cumulative tacrolimus-plus-sirolimus trough level—rather than either agent alone—may be the key determinant of immunologic outcomes comparable to standard triple therapy in kidney transplantation. Maintaining a combined trough level ≥ 11.6 ng/mL may provide the safest immunosuppressive coverage, while 8.5–11.6 ng/mL could be considered in lower-risk or dose-reduction settings; concentrations below 8.5 ng/mL within the first post-transplant year appear to carry a markedly increased rejection risk. We also observed a possible nephrotoxic synergy between sirolimus and higher tacrolimus exposure (≥7.0 ng/mL), underscoring the need for careful trough-level optimization, particularly in recipients with pre-existing renal dysfunction. Prospective randomized controlled trials are warranted to test combined mTOR inhibitor and reduced-dose CNI therapy—especially in immunologically high-risk recipients—where maintaining cumulative trough levels above approximately 11.6 ng/mL may provide effective immunosuppression while limiting nephrotoxicity.

## Figures and Tables

**Table 1:** Demographics and baseline clinical characteristics.

Variables	Total (N = 8027)	Standard ^a^ (N = 7791)	Early Conversion ^b^ (N = 236)	*p*-Value
Mean age, years	45.5 ± 13.9	45.4 ± 13.9	49.2 ± 12.2	<0.001
Male sex	4650 (57.9%)	4510 (57.9%)	140 (59.3%)	0.70
Body mass index, kg/m^2^	23.0 ± 28.8	23.0 ± 29.2	22.7 ± 3.8	0.44
Diabetes	1651 (20.6%)	1586 (20.4%)	65 (27.5%)	0.009
Pre-transplant PRA class I, %	13.2 ± 25.8	13.0 ± 25.7	17.5 ± 29.2	0.061
Pre-transplant PRA class II, %	12.2 ± 25.4	12.1 ± 25.2	15.8 ± 28.1	0.1
HLA mismatch	3.3 ± 1.5	3.3 ± 1.5	3.4 ± 1.4	0.26
Desensitization	171 (14.6%)	140 (18.0%)	31 (13.1%)	0.54
Donor age, years	44.5 ± 13.3	44.5 ± 13.3	45.8 ± 12.1	0.11
Preemptive transplantation	1454 (18.1%)	1403 (18.0%)	51 (21.6%)	0.18
Induction				
No induction	273 (3.4%)	258 (3.3%)	15 (6.4%)	<0.001
Basiliximab	6151 (76.6%)	6001 (77.0%)	150 (63.6%)	<0.001
Anti-thymocyte globulin	1601 (20.0%)	1530 (19.7%)	71 (30.1%)	<0.001
ABO incompatible	1184 (14.8%)	1154 (14.8%)	30 (12.7%)	0.42
Pre-transplant B flow, positive	261 (3.3%)	250 (3.2%)	11 (4.7%)	0.33
Pre-transplant T flow, positive	212 (2.6%)	207 (2.7%)	5 (2.1%)	0.69
Pre-transplant DSA, positive	916 (11.4%)	880 (11.3%)	36 (15.3%)	0.08
BK virus at 3 months post-transplant, positive	733 (9.1%)	693 (8.9%)	40 (16.9%)	<0.001

Categorical variables are reported as counts (percentages). Continuous variables are reported as means ± standard deviations. Abbreviations: PRA, panel reactive antibody; HLA, human leukocyte antigen; DSA, donor-specific antibody. ^a^ the standard group indicates maintenance tacrolimus and MMF; ^b^ the early conversion group included patients converted from MMF to sirolimus within 3 months post-transplant and maintained on sirolimus for at least 1 year.

**Table 2:** Baseline demographic and clinical characteristics after propensity score matching.

Variables	Total (N = 1180)	Standard Group (N = 944)	Early Conversion (N = 236)	SMD
Mean age, years	48.53 ± 13.68	48.38 ± 14.02	49.16 ± 12.21	0.39
Male sex	681 (57.7%)	541 (57.3%)	140 (59.3%)	0.04
Body mass index, kg/m^2^	22.51 ± 3.76	22.48 ± 3.76	22.66 ± 3.76	0.04
Diabetes	297 (25.2%)	232 (24.6%)	65 (27.5%)	0.06
Pre-transplant PRA class I, %	17.40 ± 29.3	17.38 ± 29.3	17.47 ± 29.2	0.02
Pre-transplant PRA class II, %	15.18 ± 28.0	15.08 ± 28.0	15.77 ± 28.1	0.02
HLA mismatch	3.25 ± 1.51	3.21 ± 1.52	3.38 ± 1.45	
Desensitization	171 (14.5%)	140 (20.2%)	31 (13.1%)	0.07
Donor age, years	45.30 ± 12.87	45.19 ± 13.07	45.78 ± 12.05	0.04
Preemptive transplantation	242 (20.5%)	191 (20.2%)	51 (21.6%)	0.03
Induction				0.05
No induction	22 (1.9%)	7 (0.7%)	15 (6.4%)	-
Basiliximab	850 (72.0%)	700 (74.2%)	150 (63.6%)	-
Anti-thymocyte globulin	279 (23.6%)	209 (22.1%)	70 (29.7%)	-
ABO incompatible	170 (14.4%)	140 (14.8%)	30 (12.7%)	0.06
Pre-transplant B flow, positive	80 (6.8%)	69 (7.3%)	11 (4.7%)	0.01
Pre-transplant T flow, positive	37 (3.1%)	32 (3.4%)	5 (2.1%)	0.06
Pre-transplant DSA, positive	137 (11.6%)	108 (11.4%)	29 (12.3%)	0.04
BK virus at 3 months post-transplant, positive	230 (19.5%)	190 (20.1%)	40 (16.9%)	0.06
Rejection at 3–12 months post-transplant	45 (7.5%)	27 (2.9%)	18 (7.6%)	0.21/ 0.001 ^a^

^a^ *p*-value corresponds to the between-group comparison of rejection occurring 3–12 months after transplantation.

**Table 3:** Infection-related outcomes by treatment strategy.

Variables	Total (N = 8027)	Standard (N = 7791)	Early Conversion (N = 236)	*p*-Value
Infection-related admission	200 (16.9%)	146 (15.5%)	54 (22.9%)	0.009
PCP	26 (2.2%)	22 (2.3%)	4 (1.7%)	0.729
BK viremia (3–12 months)	74 (6.3%)	64 (6.8%)	10 (4.2%)	0.155

Abbreviations: PCP, *Pneumocystis jirovecii* pneumonia.

## Data Availability

The raw data supporting this study’s findings are available from the corresponding author on reasonable request, without undue delay or restriction.
